# Machine learning applied to X-ray tomography as a new tool to analyze the voids in RRP Nb_3_Sn wires

**DOI:** 10.1038/s41598-021-87475-6

**Published:** 2021-04-08

**Authors:** T. Bagni, G. Bovone, A. Rack, D. Mauro, C. Barth, D. Matera, F. Buta, C. Senatore

**Affiliations:** 1 Department of Quantum Matter Physics, University of Geneva, Geneva, Switzerland; 2 ESRF – The European Synchrotron, Grenoble, France

**Keywords:** Superconducting properties and materials, Characterization and analytical techniques, Imaging techniques, Experimental particle physics

## Abstract

The electro-mechanical and electro-thermal properties of high-performance Restacked-Rod-Process (RRP) Nb_3_Sn wires are key factors in the realization of compact magnets above 15 T for the future particle physics experiments. Combining X-ray micro-tomography with unsupervised machine learning algorithm, we provide a new tool capable to study the internal features of RRP wires and unlock different approaches to enhance their performances. Such tool is ideal to characterize the distribution and morphology of the voids that are generated during the heat treatment necessary to form the Nb_3_Sn superconducting phase. Two different types of voids can be detected in this type of wires: one inside the copper matrix and the other inside the Nb_3_Sn sub-elements. The former type can be related to Sn leaking from sub-elements to the copper matrix which leads to poor electro-thermal stability of the whole wire. The second type is detrimental for the electro-mechanical performance of the wires as superconducting wires experience large electromagnetic stresses in high field and high current conditions. We analyze these aspects thoroughly and discuss the potential of the X-ray tomography analysis tool to help modeling and predicting electro-mechanical and electro-thermal behavior of RRP wires and optimize their design.

## Introduction

The intermetallic compound Nb_3_Sn is an A15 phase material with critical temperature (*T*_*c*_) of about 18 K^1^ and its upper critical magnetic field (*B*_*c2*_) can reach 30 T^2^. These characteristics combined with a high critical current density (*J*_*c*_) make Nb_3_Sn the workhorse in superconducting magnets operating in the 10 T to 20 T range. Nb_3_Sn wire technology is under development since the ‘60s, but only in the last two decades the conductor’s development has made great progress thanks to the research program for the International Thermonuclear Experimental Reactor (ITER)^3^, presently under construction. Since the ‘90s, the High Energy Physics (HEP) community has taken interest in the development of Nb_3_Sn wires for post Large Hadron Collider (LHC) accelerators. The effort was led by the European Organization for Nuclear Research (CERN) and the US LHC Accelerator Research Program (LARP)^4^, and it culminates with the adoption of Nb_3_Sn wires as baseline for the High Luminosity LHC upgrade (HL-LHC)^5^, which will include two pairs of dipoles, operating at 11 T, and 24 quadrupoles based on Nb_3_Sn. Presently, CERN is investigating various design for a new proton-proton collider with an energy of 100 TeV in the center of mass, the so-called Future Circular Collider (FCC)^6^. The maximum energy of a circular collider is determined by the maximum magnetic field of the dipole magnets and by the radius of the accelerator. Assuming a circumference of 100 km the dipole magnetic field should be about 16 T to achieve the target value of energy. Even though Nb_3_Sn wire technology has largely improved over the years, FCC requires new steps forward in Nb_3_Sn technology to achieve 16 T magnetic field in its dipoles^7^. Key factors are a high non-Cu critical current density, i.e. *J*_*c*_ > 1500 A/mm^2^ at 16 T and at 4.2 K, tolerance to mechanical loads and good thermal and electrical stabilization in the form of high purity copper (Cu) surrounding the superconducting sub-elements. Recently, four different dipole designs were developed for FCC in the frame of the Horizon 2020 European Circular Energy-Frontier Collider (EuroCirCol) study^8^: Cosine-theta^9^, Block-Coil^10^, Common-Coil^11^ and Canted Cosine-Theta^12^. The analyses performed on these designs highlight the large mechanical loads generated by electromagnetic forces on the Nb_3_Sn wires reaching peak stress of 150–200 MPa at 16 T^13^. This large stress may lead to the degradation of the electrical transport properties of the brittle Nb_3_Sn sub-elements and, for this reason, the electro-mechanical properties of Nb_3_Sn wires are a fundamental factor for the FCC dipoles’ design.

*J*_*c*_ in Nb_3_Sn depends to the applied mechanical loads and its variation has two regimes: one is reversible, i.e. the wire does not present permanent *J*_*c*_ reduction after unloading the applied force, and the other is irreversible, i.e. above a certain applied stress limit, the *J*_*c*_ degradation becomes permanent. The reversible regime has been intensively studied^14, 15^ whereas the irreversible degradation was less investigated, mostly in relation with the ITER project^16^ and more recently in the frame work for the EuroCirCol project^17, 18^ and HL-LHC^19^. In the latter publication, it has been shown that the temperature selected for the Nb_3_Sn activation heat treatment is fundamental for the irreversible limit, but, despite the remarkable collection of measurements, the authors were not able to link the phenomenon to the internal properties of the wires and unveil the mechanism behind the degradation. With the present research we offer to the community a new powerful tool to investigate the internal features of Nb_3_Sn wires at micro-meter level. This will allow to build extremely accurate electro-mechanical models considering one of the key factors in wires degradation, which is the Kirkendall voids^20–22^ generated during the activation heat treatment. In fact, the voids degrade the microstructural homogeneity^20^ and cause local stress concentration, which act as nucleation point for crack’s formation.

Therefore, the subject of the present study is the presentation of the tool and its first analysis of voids in Restacked-Rod-Process (RRP) Nb_3_Sn wires. RRP is a type of Internal-Tin Nb_3_Sn wire, and it is considered to be one of the most promising technology for future application in high-field accelerator magnets mainly because of the record *J*_*c*_ achieved^23^. Five different RRP sample wires have been analyzed at the European Synchrotron Radiation Facility (ESRF) to investigate their internal properties. Synchrotron tomography was successfully used in the past to study and characterize the voids formation of Internal-Tin Nb_3_Sn wires^24, 25^. Furthermore, a more recent study focused on bronze route Nb_3_Sn wires^26^ was able to correlate the void morphology to the critical current degradation upon mechanical load^21^ showing that the non-destructive synchrotron X-ray tomography is extremely effective to study Nb_3_Sn voids. Our analysis tool is the combination of micro-tomographies with an unsupervised machine learning algorithm, which is able to autonomously isolate, reconstruct and analyze the voids. In the studied wires, the tool detected two type of voids: in the high-purity copper matrix and in the Nb_3_Sn sub-elements. Some of the voids in the Cu-matrix are related to the presence of gas volumes generated during the wire production whereas other are Kirkendall voids^27^, which are due to the Sn diffusion in the Cu-matrix^24^. The latter are thus indicators of a degraded electro-thermal stability of the wire. Differently, the voids in the sub-elements are detrimental for electro-mechanical performance of the wire. The accurate 3D geometrical characterization of the wires internal features is a fundamental step along the path of mechanical and electro-thermal enhancement of RRP wires.

The paper first discusses the main properties of the RRP Nb_3_Sn samples used in the analysis. The second part is devoted to X-ray tomography performed at the ESRF. The last section is dedicated to the detection and analyses of the voids and their implications.

### Samples description

**Figure 1:**
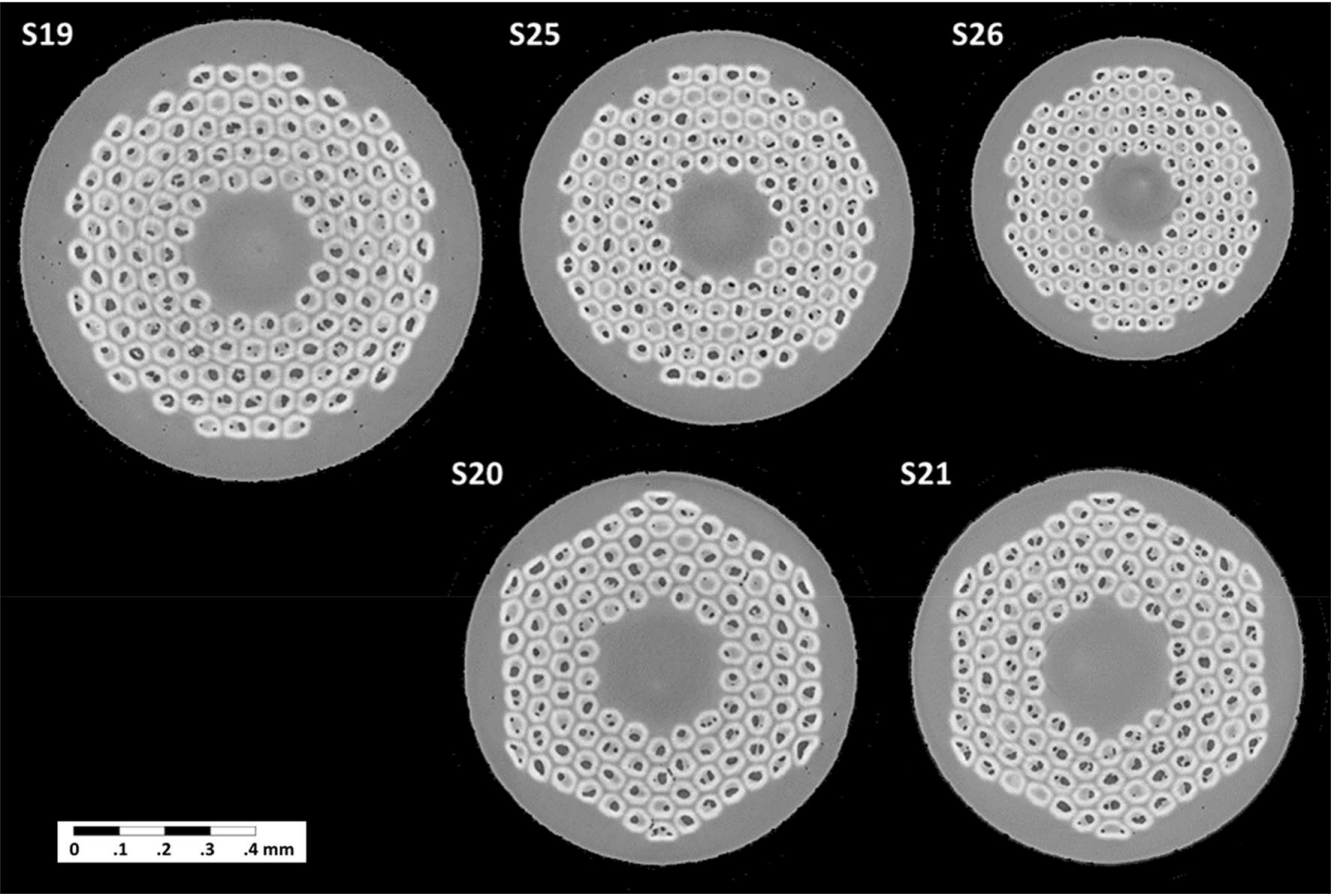
X-ray tomography cross section of the Nb_3_Sn RRP wires tested at the ESRF facility.

In this study, the emphasis is on the voids present in Nb_3_Sn RRP wires. Two types of wire configurations were investigated: 108/127 and 132/169. These numbers define the RRPs configuration as number of superconducting sub-elements over the total of restacked elements. Each design was used for a different study goal. 108/127 wires have been used for the 11 T dipoles^28^ and for the Low-β quadrupole magnet (MQXF) of the HL-LHC upgrade^29^, at a diameter of 0.7 mm and 0.85 mm, respectively. Two different versions of this design were analyzed, both 0.85 mm in diameter: S20 is Ta doped wire with normal Sn content, which correspond to a Nb:Sn molar ratio of 3.4 and S21 is Ti doped with a reduced Sn content, i.e. Nb:Sn molar ratio of 3.6. The different Nb:Sn molar ratios allowed us to study the correlation between Nb content, Sn leakage and presence of voids in the copper matrix. 132/169 wires were developed in the framework of the European Coordination for Accelerator Research and Development (EuCARD)^30^ and LARP^31^, and they have been used for studies related to FCC. The three samples are reduced Sn and Ti doped wires with different diameters. The different diameters, and consequently different sub-elements sizes, allowed us to investigate how the distribution of voids in the sub-elements is affected by the dimension of the sub-element itself. Samples characteristics and heat treatments are listed in Table 1 and samples cross sections are shown in Fig. 1.

**Table 1:** Summary of samples characteristics, Residual Resistivity Ratio (RRR) and Cu void fraction.

Sample	Diameter [mm]	Wire configuration	Nb:Sn molar ratio	Heat treatment	Cu void fraction [% of V_total_]	RRR
S20	0.85	108/127	3.4	210 °C × 48 h + 400 °C × 48 h + 650 °C/50 h (all ramps at 25 °C/h)	0.1	89
S21	0.85	108/127	3.6		0.05	274
S19	1	132/169	3.6	210 °C × 48 h + 400 °C × 48 h + 650 °C × 50 h (all ramps at 50 °C/h)	0.07	400
S25	0.85	132/169	3.6	210 °C × 48 h + 400 °C × 48 h + 640 °C × 50 h (all ramps at 50 °C/h)	0.02	358
S26	0.7	132/169	3.6		0.02	204

### X-ray tomography

X-ray micro-tomography has been performed at the beamline ID19 of the ESRF in Grenoble, France. Synchrotron micro-tomography is an exceptionally powerful tool to study the porosity of materials on µm scale. X-ray synchrotron radiation was already proven to be suited to study the internal features of superconducting wires in several studies^25, 32^. Furthermore the use of narrow bandwidth radiation helped us to improve the contrast between Nb_3_Sn and Cu voids^33^ and prevented beam hardening effects, which is the phenomenon that occurs when polychromatic X-ray beam passes through an object, resulting in selective attenuation of lower energy photons^34^. X-ray micro-tomography allows detecting and defining internal structure of a composite in a non-destructive manner without influencing the sample. The sample is exposed transversally to the X-ray beam and an image of the transmitted beam is recorded with a detector placed behind the rotating sample holder. In our case the sample was rotated of 360° in 0.012° steps, i.e. 30,000 projections images acquired per sample. These projections, once stacked into a three-dimensional (3D) X-ray absorption map, provide a 3D reconstruction of the sample volume with its internal characteristics. The photon energy was 89 keV and the detector had 2560 × 2160 pixels resolution with an equivalent spatial sampling of about 0.7 μm/pixel (empirically verified on the wire diameter). The final output of the measurements is a set of two-dimensional (2D) images of the measured Nb_3_Sn samples where each image corresponds to a cross-section of the wire, see Fig. 1. The length of wire that was analyzed in this way was of about 1.5 mm equivalent to 2160 images per samples.

## Results

### Void detection and analysis

**Figure 2:**
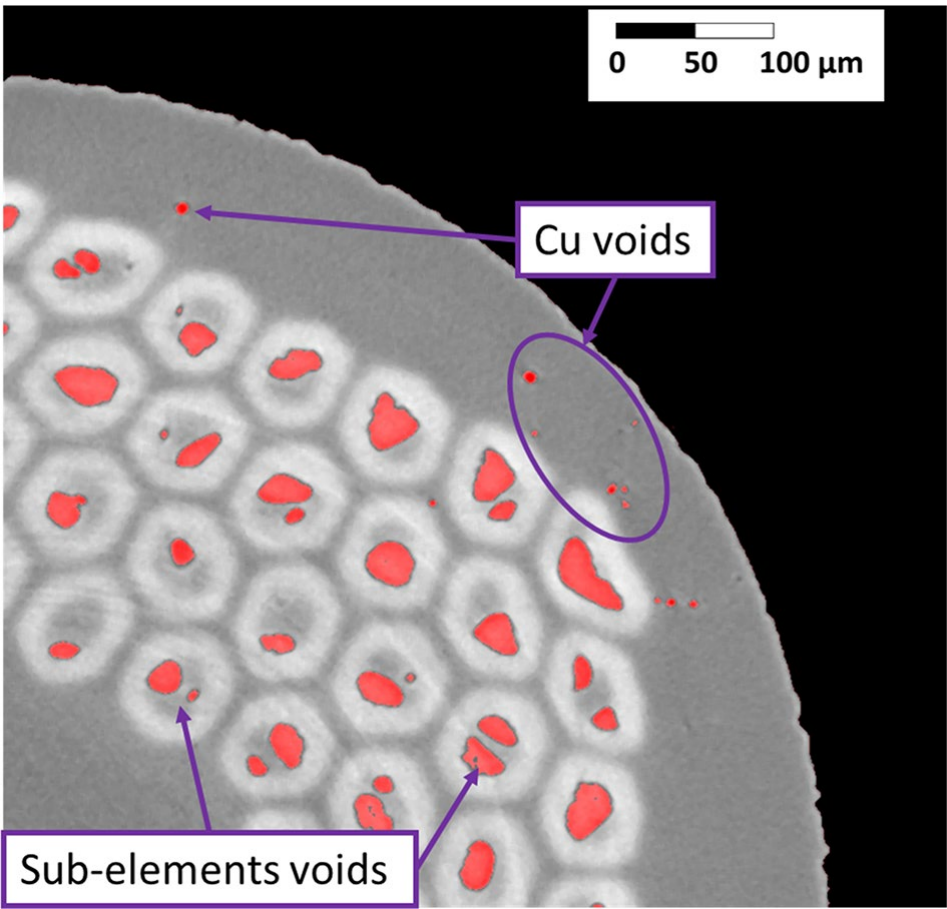
Detection of the voids in a x-ray tomography of the sample S20. Cu and sub-elements voids are in red.

The tomography post-process was begun exploiting a dedicate algorithm written in MATLAB and already tested in^21^. The MATLAB tool analyses the 2D tomography’s cross-sections one by one. To detect the voids, each pixel is compared with a color threshold that is calibrated on the slice average brightness. The voids are saved as binary maps were 1 is a void pixel and 0 is everything else. In Fig. 2, a section of an analyzed tomography is shown with the voids detected in red. Two different type of voids are visible, those located in the sub-elements and those in the Cu matrix. The MATLAB tool was not capable to separate the voids by location and thus discriminate the voids in the sub-elements from those in the Cu matrix. In case of RRPs, it is fundamental to separate Cu voids from sub-elements voids because they are generated differently, and they can be responsible for different phenomena. Therefore, we programmed an entirely new tool creating a more flexible program based on unsupervised machine learning implemented via Python.

The separation of the wire components in the tomography was done using the well-established *k*-means method^35^, an unsupervised classification algorithm developed for clustering. The underlying idea of clustering is to divide a given set of data into a specific number of groups based on certain patterns or similarities present in the data; in our case the groups (sub-elements, voids and Cu matrix) were defined based on the pixels brightness of the tomography. *k*-means algorithm divides the given set of data into *k* disjoint clusters equivalent to the groups. The analysis of an image, i.e. one slice of the tomography, can be summarized as follows: in the initial step a *k* number of centers of brightness, *c*_*k*_, are generated based on the picture brightness scale. The initial centers are arbitrarily generated by the algorithm, the operator can control the number of time the *k-*means algorithm will be run with different initial *c*_*k*_, the maximum number of iteration per run, and the relative tolerance used to declare the convergence^36^. For every pixel of the image, the Euclidean distance on the brightness scale *d*, between the centers and pixel brightness, *p(x,y)*, is calculated as:

1 $$d=\left|p\left(x,y\right)-{c}_{k}\right|$$

Then, the pixels are assigned to the nearest center based on the calculated brightness distance. When all pixels have been assigned, a new set of centers is generated using Eq. (2), in which *n*_*k*_ denotes the number of observations in the *k*^*th*^ cluster^37^. The process is repeated until the tolerance is satisfied, i.e. there are no significant changes in the centers positions.

2 $${c}_{k}=\frac{1}{{n}_{k}}\sum_{y\in {c}_{k}}\sum_{x\in {c}_{k}}p(x,y)$$

As a last step the clusters of pixels are saved. More on *k*-means algorithm can be found in^38^. *k*-means algorithm was implemented using the python scikit-learn library^39^. After applying the *k*-means algorithm, further processing was done in order to obtain as a final result three separated binary images: Nb_3_Sn sub-elements, Cu matrix and voids. Eventually, copper voids were achieved subtracting the sub-elements from the voids’ maps.

**Figure 3:**
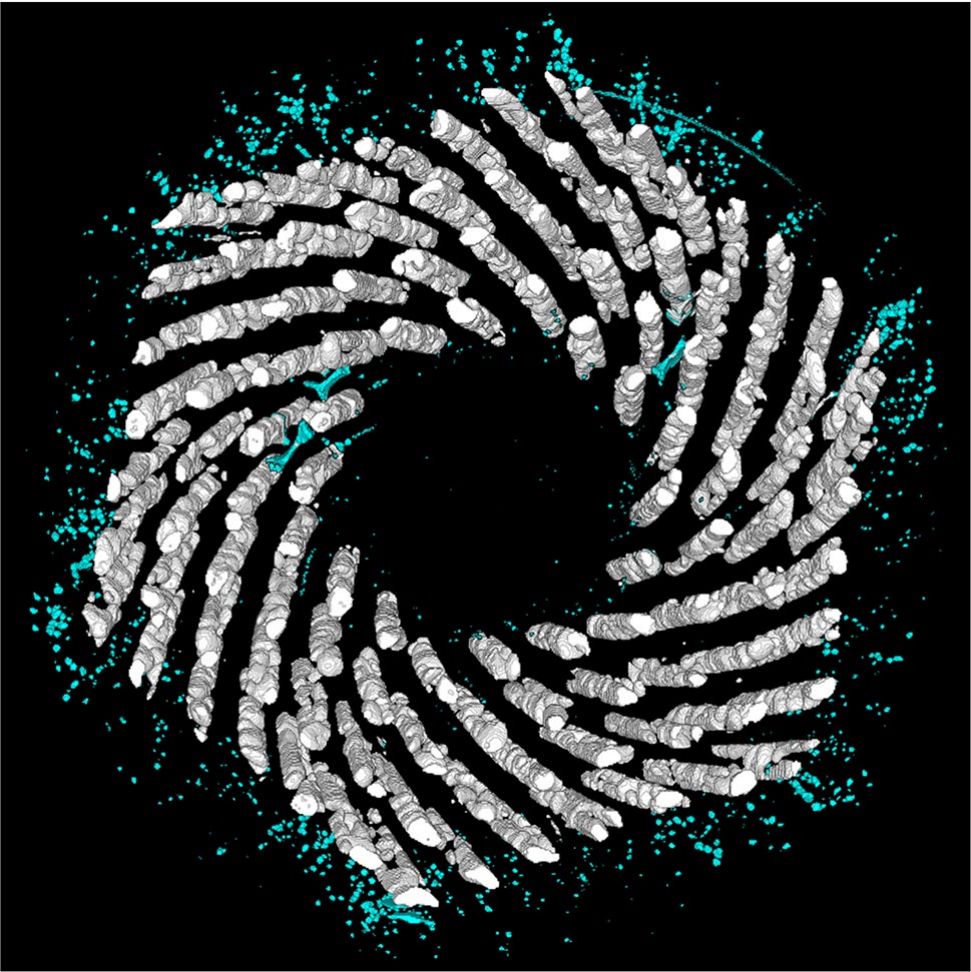
The 3D voids reconstruction of the sample S20. The voids in the sub-elements are white while the copper voids are light blue. The image was generated by using the Fiji (Fiji is Just ImageJ) distribution of ImageJ^40^. [ImageJ-1.53c https://imagej.net/ImageJ].

**Figure 4:**
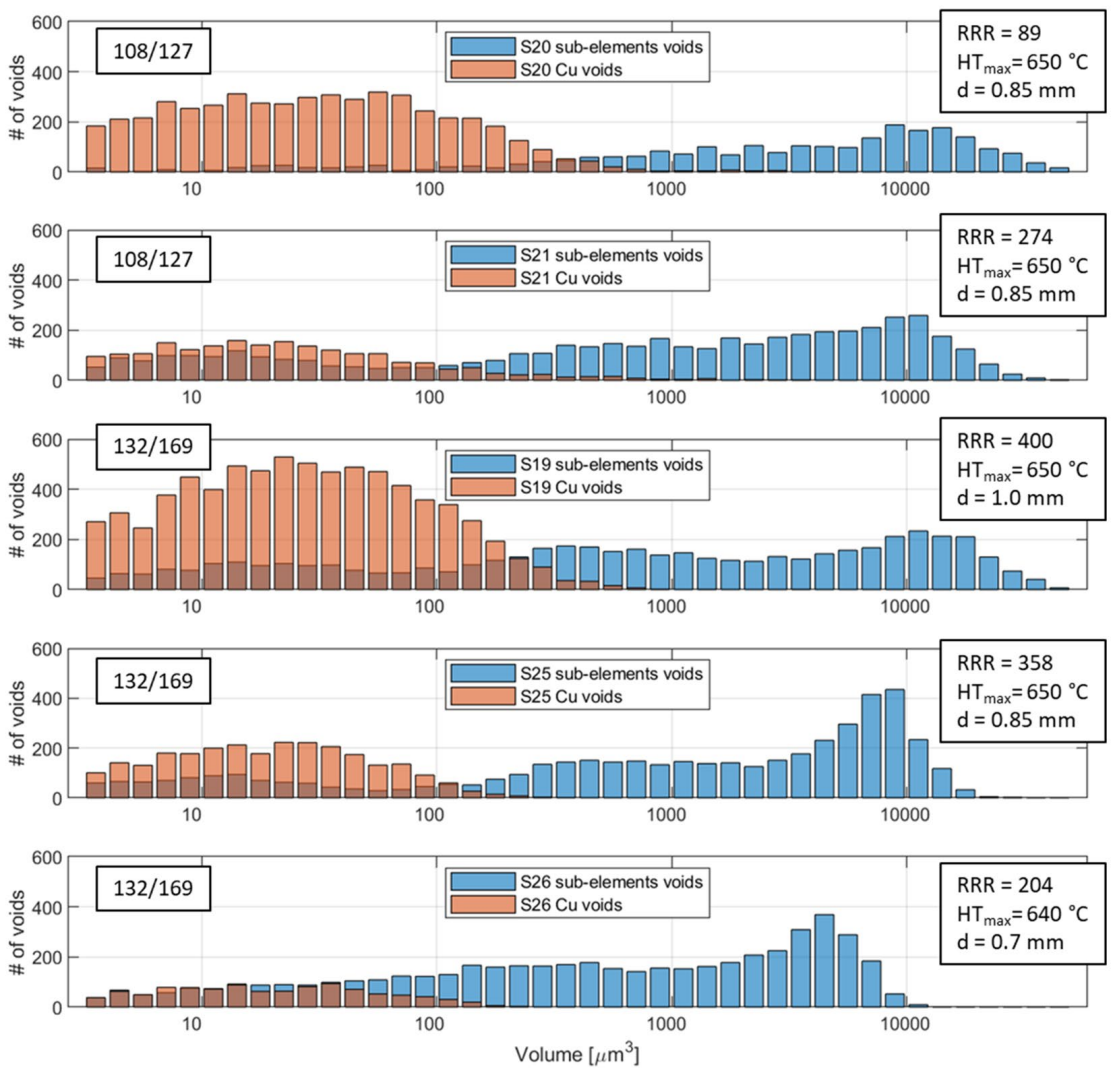
Distribution of the wires sub-element and copper voids as function of their volume.

The 3D reconstruction of the voids for the wire S20, differentiated by type, is shown in Fig. 3. The larger volumes are in the sub-elements, while the small voids are in the copper shell and core, some copper voids are among the sub-elements and they are indication of Sn pollution. The voids’ volume was calculated from the 3D voids reconstruction knowing the pixel/volume ratio. By separating the voids by type, the distribution as function of volume shows that the majority of copper voids has a volume smaller than sub-elements voids, but they can be far more numerous. Fig. 4 reports this and shows the data for all the examined wires. In particular, the strong variability of the Cu voids distributions among the samples, in comparison to the more homogeneous sub-elements voids distribution, highlights that the sub-element voids and the Cu voids have a different origin.

### Copper voids, Nb:Sn ratio and correlation with the residual resistivity ratio

From the direct observation of S20 in Fig. 3, it is evident that the morphology of copper and sub-elements voids is different. The sub-elements voids have larger dimensions than Cu voids and they are present in all sub-elements, whereas Cu voids are mostly concentrated in the outer copper shell and in the central core with few larger voids between the sub-elements. Differently from S20, the 3D reconstruction of S21 had copper voids only in the outer copper and few in the central core. Therefore, two types of Cu voids can be observed in S20: small voids in the Cu shell or core, generated by the coalescence of micro defects trapped during the wire assembly, and larger Kirkendall voids between sub-elements, due to Sn pollution generated by Nb barrier failures. The former type is present in all the wires and depends on the assembly procedure, the latter depends on different factors as Nb barrier thickness and heat treatment. Since they are independent, we are going to discuss the two types of voids separately, starting from the Kirkendall voids generated from Sn diffusion.

As said, the analyzed 108/127 wires have similar design but different Nb:Sn molar ratio. It has been shown in^41^ that normal Sn content RRP wires have typically lower Residual Resistivity Ratio (RRR) than reduced Sn content wires. Our RRR measurements are in agreement with this result, S20 has RRR = 89 while for S21 has RRR = 274, indicating a higher Cu purity for the latter.

**Figure 5:**
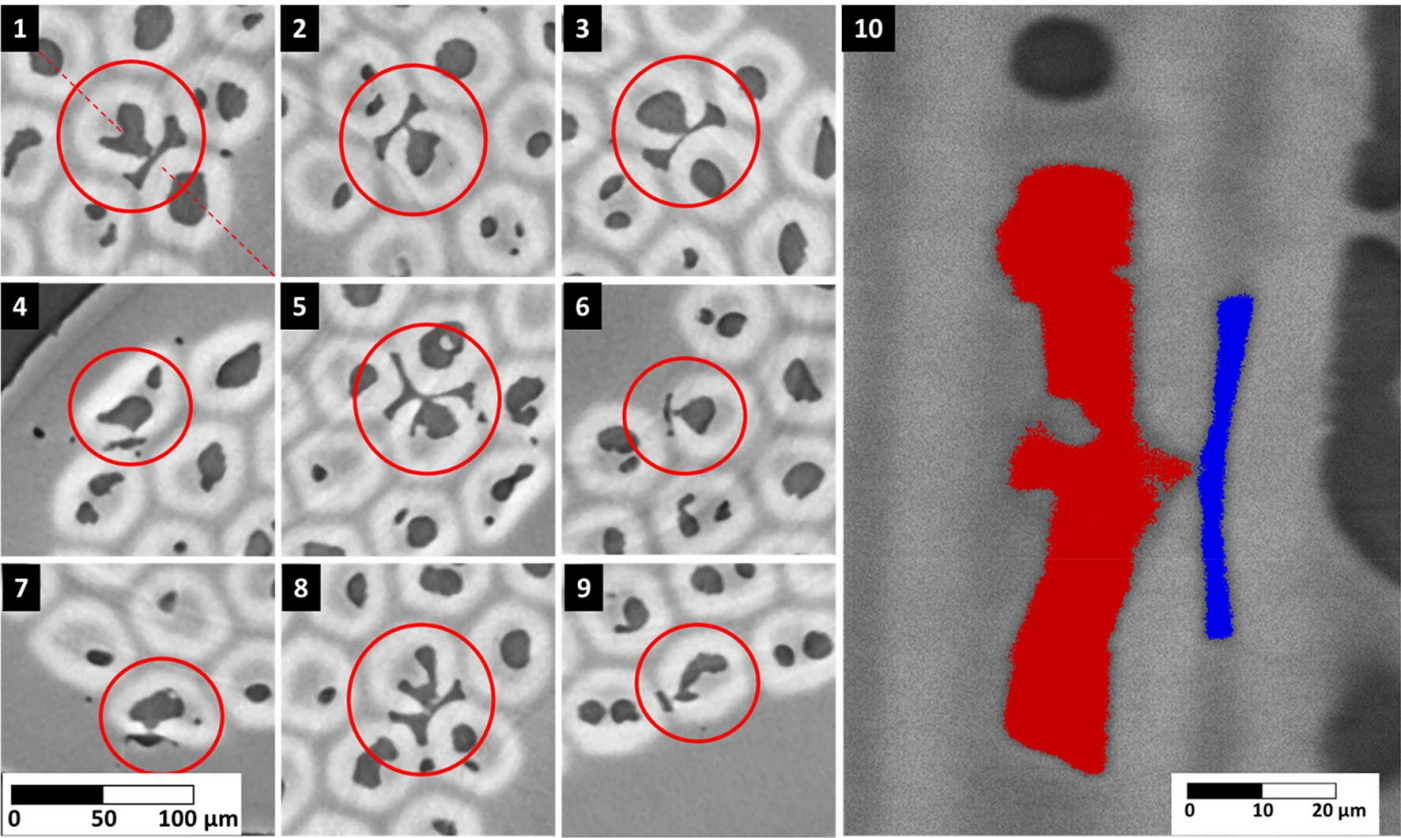
Collection of Cu voids generated by Sn diffusion due to damaged Nb barriers in S20. The void in the sub-element is connected to the Cu void crossing the Nb_3_Sn ring at the Nb barrier rupture. Pictures 1 to 9 are transversal cross sections of the wire. Picture 10 shows the longitudinal cross section of void #1 cut along the red dashed diagonal shown in picture 1. The void is highlighted in red inside the sub-element and in blue in the Cu matrix. The images are obtained from the 3D reconstruction of the X-ray tomographies.

Since low RRR measurement can be an indication of the Sn presence in the Cu matrix, a manual investigation of Nb barriers macroscopic disruptions was performed on the wires’ tomography looking for barrier failures and related Kirkendall voids. Studying the tomography of S20, several barriers’ ruptures were detected. In presence of Nb barrier breakages, the voids extend from the sub-element to the Cu matrix crossing the Nb_3_Sn ring, allowing visual detection of the phenomenon. In Fig. 5, a collection of barrier disruptions is shown. Thanks to the 3D reconstruction of the tomographies, it is possible to appreciate the exact spot where the barrier fails, generating the Sn leakages, in both transversal and longitudinal directions.

**Figure 6:**
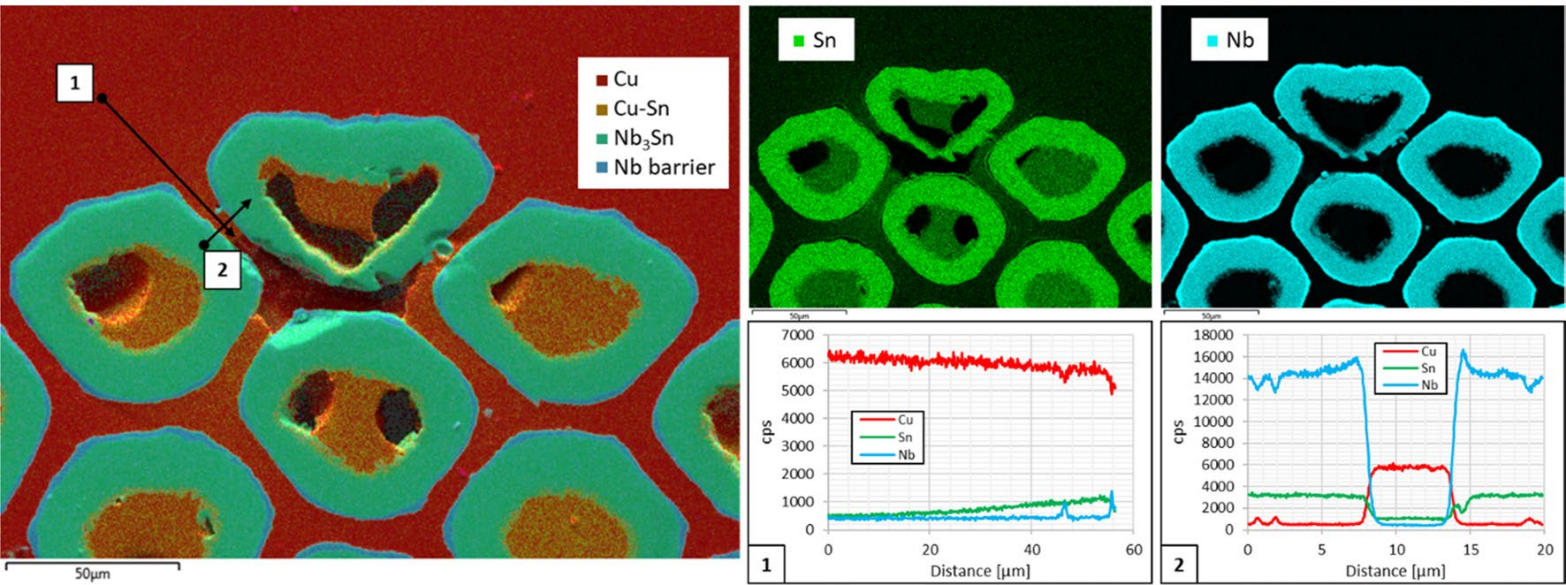
SEM–EDX analysis of a cross section of S20. The analysis was focused on the void located in the Cu matrix. The image on the left shows the EDX map of the cross section and every color indicate a different element of the wire. The EDX maps of Sn and Nb are the top right pictures. The Sn map shows the diffusion of the material outside the sub-elements. The EDX line scan 1 and 2 show the gradient of the Sn diffusion from the void into the Cu matrix.

In general, it is expected that heavily deformed sub-elements are more prone to barrier failure. The 108/127 design has a hexagonal pattern of the sub-elements and, during the drawing process, the sub-elements in the corners are more subject to deformation stress, as can be observed in the cross sections reported in Fig. 1. Despite the limited statistics, the results show that about 20% of the broken barriers are in the corners of the wire, indicating that the 108/127 design can be particularly susceptible to this phenomenon. This qualifies our tool to identify problematic sub-elements in the wire arrangement and can be of use to manufacturers in order to optimize or tailor the sub-element barrier thickness depending on the sub-elements arrangement. As final evidence of the presence of Sn in the Cu matrix a SEM–EDX analysis was performed on five S20 cross sections. In one of the cross-section was possible to locate a Cu void in contact to one of the highly deformed sub-elements, see Fig. 6. The EDX analysis shows presence of Sn in the Cu and, in addition, the Sn concentration increased approaching the void.

Both tomography and SEM–EDX analysis, performed on S21 samples, were not capable to detect Nb barrier ruptures. These results confirm that a Nb:Sn ratio of 3.4 is more prone to Sn diffusion in the Cu matrix at this heat treatment conditions and that a ratio of 3.6 is recommended, in agreement with the results of^42^ and^41^. As a further step in the improvement of our analysis tool, we plan to automatize the identification of the broken barriers by implementing advanced machine-learning and deep-learning technologies, such as object detection^43^ and semantic segmentation^44^.

**Figure 7:**
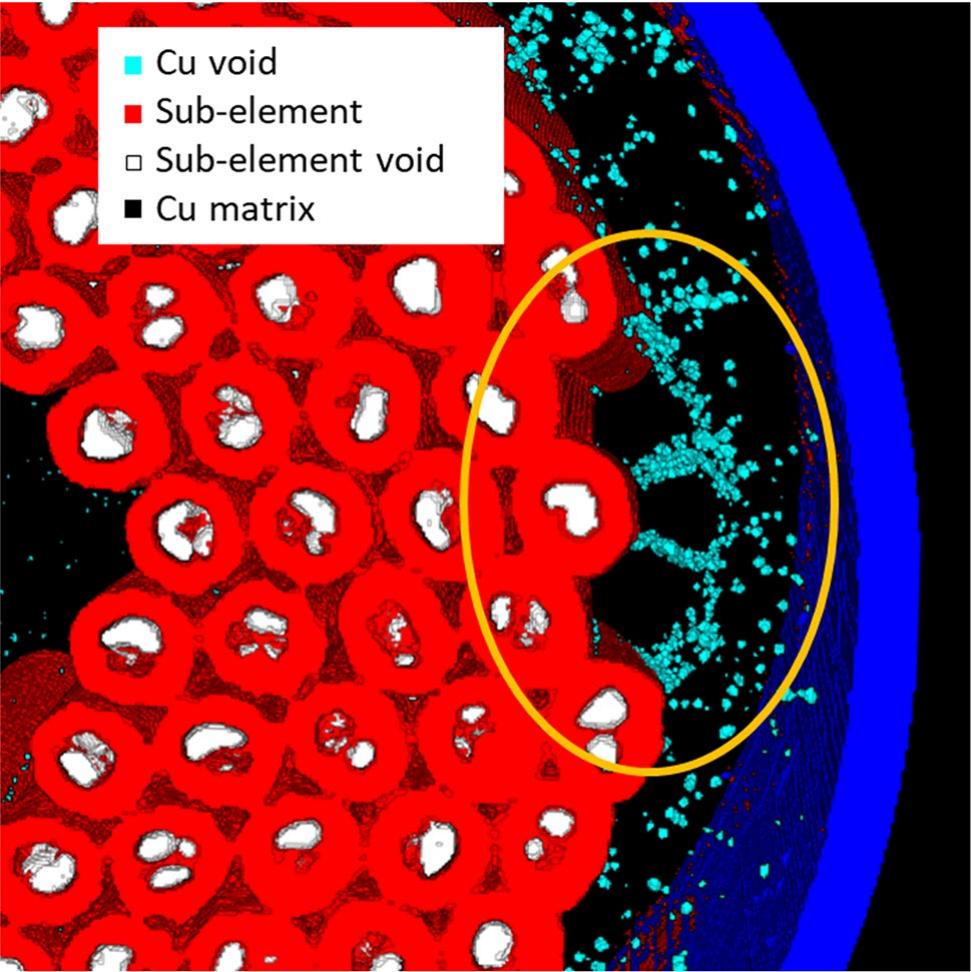
The 3D reconstruction of sample S19. The sub-elements and their voids are in red and white, respectively. The blue wall represents the wire edge, while the Cu voids are in light blue. The orange circle underlines three sets of Cu voids formed around hexagonal Cu rods used to fil the space between the sub-elements and the outer copper tube. The image was generated by using the Fiji distribution of ImageJ^40^. [ImageJ-1.53c https://imagej.net/ImageJ ].

The analysis tool highlights that even with high purity Cu matrix, i.e. high RRR values (> 150), all the analyzed samples show presence of Cu voids, see Table 1. Furthermore looking at Fig. 4, it is clear that the Cu purity is not directly linked to the number of Cu voids since S19, the wire with the most Cu voids, is the wire with the highest RRR. In this case the voids are generated during the wire production. In the final steps of the wire assembly, sub-elements and copper hexagons are stacked inside the high purity Cu tube, which constitutes the outer layer of the wire. In this process Cu rods can be added to fill the empty spaces between hexagons and the Cu tube. Because of the unavoidable asperities at the surface of the components being assembled, defects can be incorporated between these components. Some of them will disappear during the subsequent deformation but part of them will stay trapped between the copper components. X-ray tomography performed on the wires before the activation heat treatments were not able to detect such defects. Therefore, as we suspected, these defects are significantly finer at this stage and they migrate and coalesce during the reaction heat treatment forming small voids in the copper matrix. Fig. 7 shows Cu voids and sub-elements in the 3D reconstruction of S19. In the highlighted area, Cu voids are homogeneously distributed around hexagonal volumes proving that Cu filler rods are the source of these type voids. These Cu voids do not affect the purity of the Cu but an excessive number could change the Cu matrix morphology varying the conductive path inside the matrix and therefore influencing the wire electro-thermal stability. During the SEM–EDX analysis, none of these Cu voids were visible in the Cu matrix. The most probable cause is the SEM samples preparation. To obtain a flat and clean surface, the wire is polished with progressively smoother polishing cloths, during this process the soft material can be easily deformed hiding the Cu micro-voids. For this reason, it does not surprise that this phenomenon was not reported so far for RRP wires.

### Sub-element voids characterization

The sub-element voids are generated during the wire heat treatment, necessary for the formation of the Nb_3_Sn phase. As listed in Table 1, the heat treatment has three plateaus at different temperatures, at 210 °C, 400 °C and 640–650 °C. The sub-element voids begin to form after the first plateau, above 210 °C, coinciding with the appearance of a Cu-Sn phase^45^. These Kirkendall voids, due to the diffusion of Sn in Cu and Nb, grow by coalescence during the heat treatment steps. They reach the final dimensions during the formation of the Nb_3_Sn phase, where the majority of Sn bond with the Nb leaving in the sub-elements core large voids in a Cu-Sn solution. More on the sub-element voids formation is described in^24^.

S19, S25 and S26 are three 132/169 RRP wires with different diameters. The heat treatments of the wires are tailored on the wire design as well as the sub-elements dimensions. In particular, the final temperature plateau and its duration are optimized depending on sub-elements size. The temperature and duration are empirically defined by the manufacturer to maximize the A15 reaction and the *J*_*c,*_ without reducing the RRR. Previous studies showed that heat treatments longer than 50 h with a temperature higher than 670 °C stimulate the Sn diffusion increasing the probability of Sn leakage into the Cu stabilizer^46^. On the other hand, in case of lower temperature the Sn may not diffuse enough causing worse performances at high fields^47^. With these considerations, RRP can only exploit a limited temperature window between 640 and 665 °C to optimize the wire properties.

The three samples have different diameters, and because they have the same configuration, this difference is reflected by the sub-element size. For this reason, the average diameter of the sub-elements, measured from the 2D tomography, shows a linear dependence on the wire size, see Table 2. Due to the deformation of the hexagonal sub-elements during the production process, the sub-elements shape can vary within the same wire impacting the dimensions of the voids as well. Furthermore, the sub-elements physically limit the maximum dimension of their voids, so the sub-elements’ voids must scale as function of the wire diameter.

**Table 2:** Characteristics of the voids in the sub-elements for samples S19, S25 and S26.

Sample	Sub-element diameter [µm]	Sub-element void fraction [% of V_total_]	Voids max. volume [µm^3^]
S19	55 ± 5	4.2	66,300
S25	47 ± 5	4.3	27,600
S26	38 ± 4	4.0	19,900

**Figure 8:**
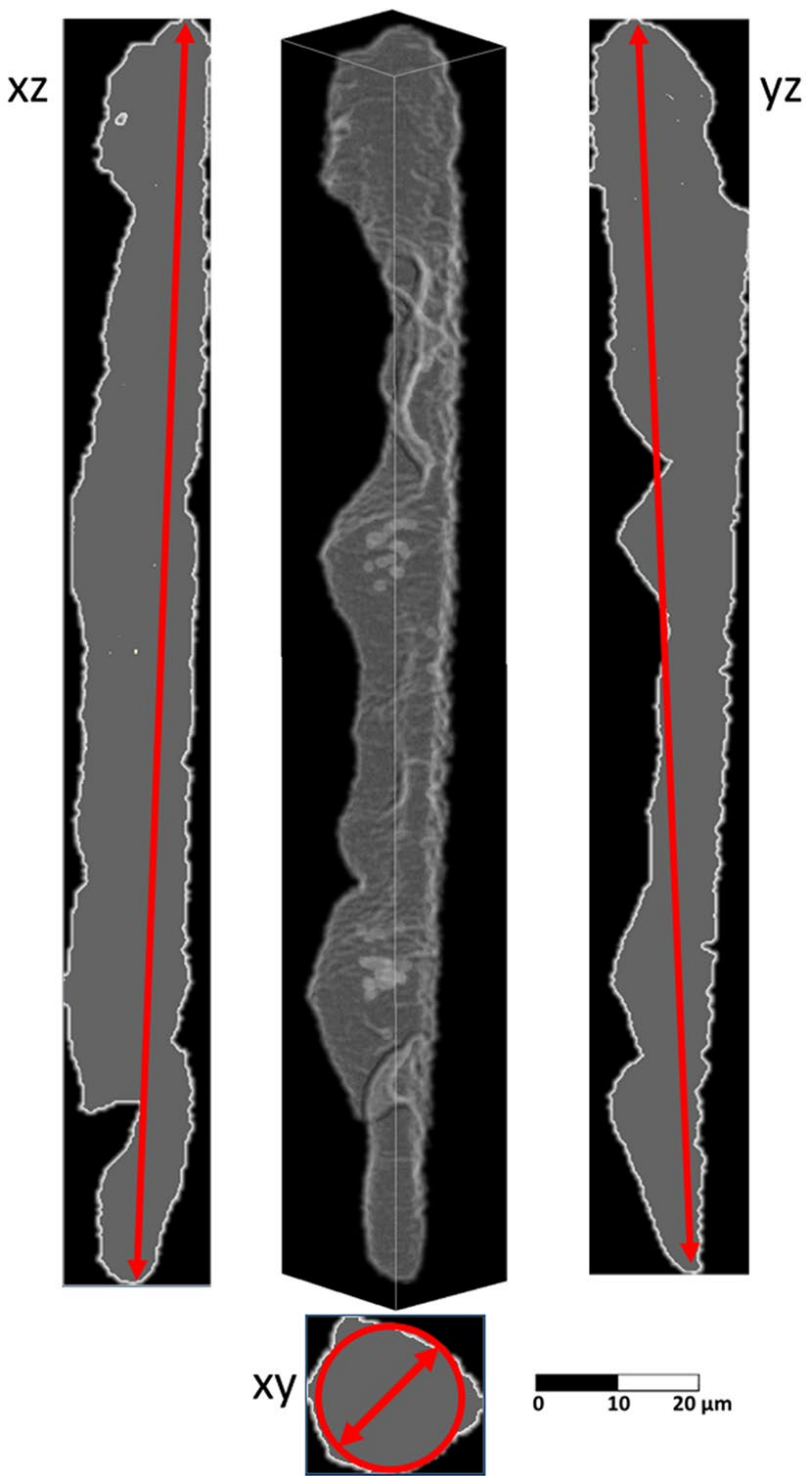
The 3D reconstruction of a sub-element void of S19 sample. The projections of the void are shown in grey. The length of the voids is estimated using the major axes of the projections on XZ and YZ planes, while the void diameter was calculated from the circle equivalent to the projection on the plane XY. The image was generated by using the Fiji distribution of ImageJ^40^. [ImageJ-1.53c https://imagej.net/ImageJ ].

To verify this relation, we used our analysis tool, which provides the voids volumes and their center of mass, allowing to uniquely locate their position in the sample. Sub-elements voids are strongly irregular, therefore, to assign them a geometrical description, we decided to approximate them to simple ellipsoids. In such approximation, the major axis of the ellipsoid is equivalent to the void length whereas the minor axis of the ellipsoid is the void diameter. These dimensions have been calculated using the projections of the voids to the XZ, YZ and XY planes, as shown in Fig. 8. The void length has been defined as the average of the major axes of the XZ and YZ projections, while the diameter is calculated from the circle which has the equivalent area of the void XY projection.

**Figure 9:**
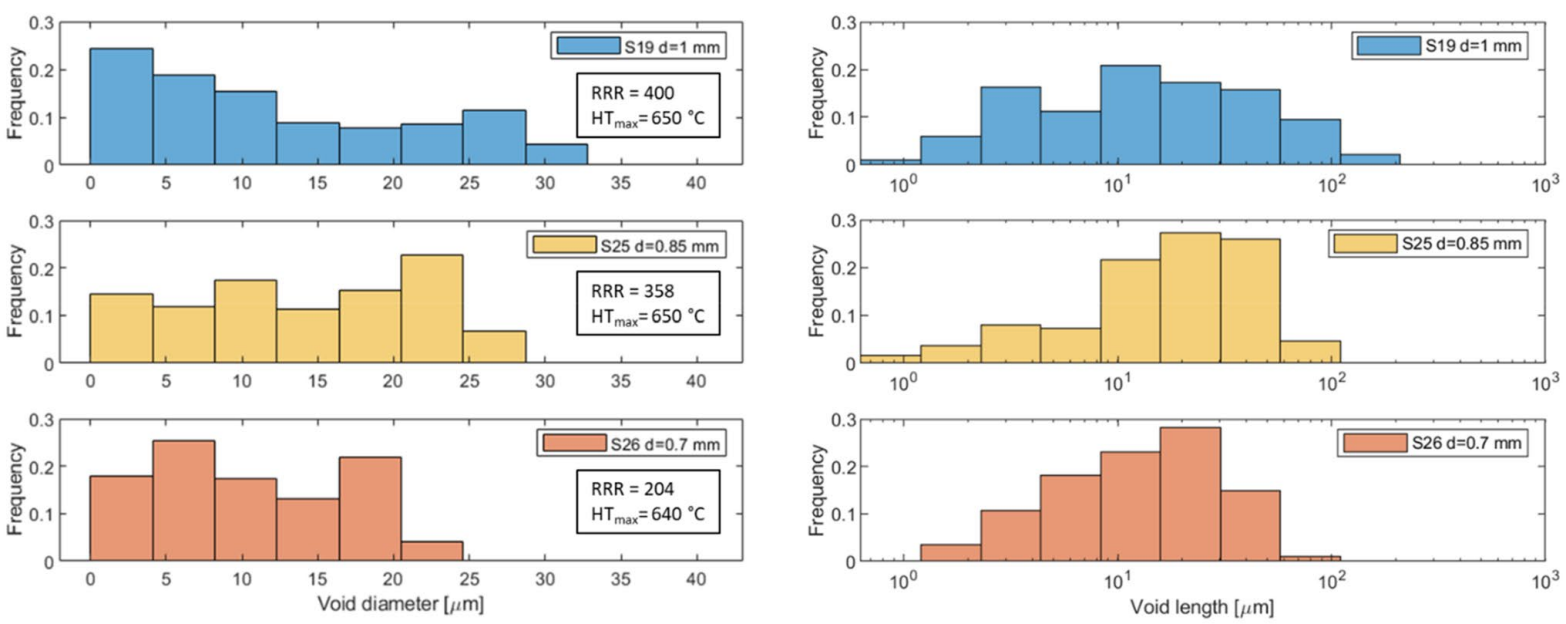
Frequency of the sub-elements voids as function of diameter (left) and length (right) for samples S19, S25 and S26.

The resulting frequency distributions of sub-elements voids’ lengths and diameters as function of the wires’ dimension is shown in Fig. 9. It is important to underline that the voids at the top and bottom of the analyzed samples can be incomplete. The tomography virtually slices the wire cutting off the voids at the edges, and consequently, these shortened voids have reduced volumes and dimensions. Therefore in Fig. 9 the left tails of the distributions are overestimated by this intrinsic generation of incomplete voids. The distribution shows the dependency of the voids’ diameter and length from the wire size, showing that voids maximum volume scale with wire and sub-elements size and that the average voids length is approximately one order of magnitude less than the analyzed sample length. In addition, the fraction of the sample occupied by the sub-element voids should be constant, and in fact, the calculated void fractions are between 4.0% and 4.3%, with the highest percentage in sample S25, see Table 2.

The difference in voids distribution among the three 132/169 RRP wires shows the complexity of the problem. The three samples have the same design and are in scale for sub-elements dimensions, but because of their differences in internal features and heat treatment, they have different voids distribution, thus they cannot be considered mechanically equivalent, i.e. one wire cannot be used as mechanical model scale for the others^48^. In this context a simple mechanical model based only on the sub-elements design cannot provide reliable results, because the sub-elements voids cannot be neglected, as they can act as nucleation point for the cracks in Nb_3_Sn^21^. Therefore to study and enhance the mechanical properties of RRP wires, a more complex mechanical model, capable to include the Kirkendall voids in the sub-elements, is necessary. Our geometrical characterization of the voids makes possible to overcome this issue providing the effective internal features of RRP wires calculating position, orientation, and dimensions of the voids and if needed of the sub-elements. This capability will unlock new strategies for pushing forward the development of RRP Nb_3_Sn wires obtaining wires with enhanced tolerance to stress by adjusting, for example, the heat treatment in order to tailor the voids distribution without neglecting the effects on electro-thermal properties and critical current.

## Discussion

The information collected using the presented analysis tool raises new questions on voids formations, sub-elements deformations and barrier failures, which are a clear indication of the potential impact such instrument can have on the comprehension of RRP wires properties. A possible application could be to use the tool to quantify the impact of voids on the electro-mechanical behavior of the wire and, as consequence, to improve the mechanical limits of RRP Nb_3_Sn wires. As matter of fact a clear quantitative correlation between voids and mechanical limits was obtained for Bronze route wires^21^ while for the other types of internal tin wires, such as RRPs, the correlation has yet to be demonstrated. It was shown that varying the dopants (Ti or Ta) and the temperature of the heat treatment final plateau (600–750 °C), the axial irreversible limit of RRP wire changes^49^. Using the combination of x-ray tomography and unsupervised machine learning algorithm would be possible to completely characterize Nb_3_Sn wires and quantify the impact of the voids variation on the irreversible limit due to the different heat treatments. The software detects and separates sub-elements, copper matrix and voids, and calculates their position, volume, orientation, and dimensions. Hence, the tool quantifies the wire characteristics paving the way for Finite Element Models (FEM) at sub-elements level, and further optimization of electro-mechanical properties of Nb_3_Sn wires.

Furthermore, even if the tool was developed to analyze Nb_3_Sn RRP wires, it will have a significant impact on different technologies. This algorithm is ideal to characterize the wires whose production process involves a synthesis reaction or sintering heat treatment between precursors, which can cause the generation of voids or deformations of the superconducting filaments. The strength of the unsupervised machine learning is the ability to separate the elements that constitute a wire regardless of the materials involved, while the reconstruction program can easily be adapted to the wires components. In particular, the tool can provide support in the advancement of Powder in Tube (PIT) superconducting wires. In Bi-2212, voids have almost no effect on the mechanical behavior of the wire^50, 51^, on the other hand bridging or bonding of the filaments was related to the heat treatment process and proven to be partially responsible for low *J*_*c*_ and *n*-value^52, 53^. The analysis tool could easily map the filaments bridges allowing to quantify the number of filaments intergrowths and their impact on *J*_*c*_. Others superconducting wires based on powder metallurgy, as Iron-based^54^ and MgB_2_ wires^55^, could also benefit from tomography analysis by analyzing, for example, the interaction between the metal sheath and the superconducting phase. The technique can also be applied in in-situ MgB_2_ wires for the investigation of the voids formation, due to volume contraction during the synthesis process, which could be detrimental for *J*_*c*_ and mechanical limits^55–57^. Finally, the tool is not limited to study single wires, more complex system can also benefit from its use. The analysis of voids and cracks distribution of Nb_3_Sn Rutherford cables after load test^58^ can provide precious information of the most sensible area of the cable and how to improve the cable design. While the study of the deformations of the wires in cable in conduit conductors (CICCs) for fusion application^59^ can offer new solutions for the wires twisting pattern in order to optimize such conductors.

## Conclusion

In this paper we propose a new tool for the study of the internal features of RRP Nb_3_Sn wires. The combination of X-ray micro-tomography and unsupervised machine learning algorithms is used to analyze two 108/127 wires with different Sn content and three 132/169 wires with different diameters. The unsupervised machine learning algorithm allows to differentiate sub-elements voids from Cu voids providing their 3D reconstruction and geometrical characterization.

108/127 samples have been used to study the variation of Cu voids depending on the Sn content. In case of normal Sn content, Kirkendall voids were generated by Sn diffusion resulting from ruptures in the Nb barriers. Sn pollution in the Cu matrix results in poor electro-thermal stability of the wire. In addition, the analysis underlies that, unsurprisingly, the barrier failures are more frequent in highly deformed sub-elements. In case of the reduced Sn wire, i.e. Nb:Sn molar ratio of 3.6, Sn contamination was not detected in the Cu matrix. Nevertheless, a large number of Cu voids was observed in all the samples, these voids were generated by defects trapped between the different constituents of the Cu matrix during the production process and they do not have a negative impact on the copper RRR.

The three samples of different diameters but same design (132/169) have been used to study the voids distribution and dimensions. The voids were morphologically similar, and the maximum voids volume scales with the diameter of the sub-elements. On the other hand, the distribution of the voids dimensions presents differences which do not allows to directly extrapolate electro-mechanical properties from similar wires. The analysis demonstrates the importance of the accurate description of a wire’s internal characteristics and the effectiveness of our analysis technique for studying RRP Nb_3_Sn wires.

## Methods

### RRP Nb_3_Sn wire technology

**Figure 10:**
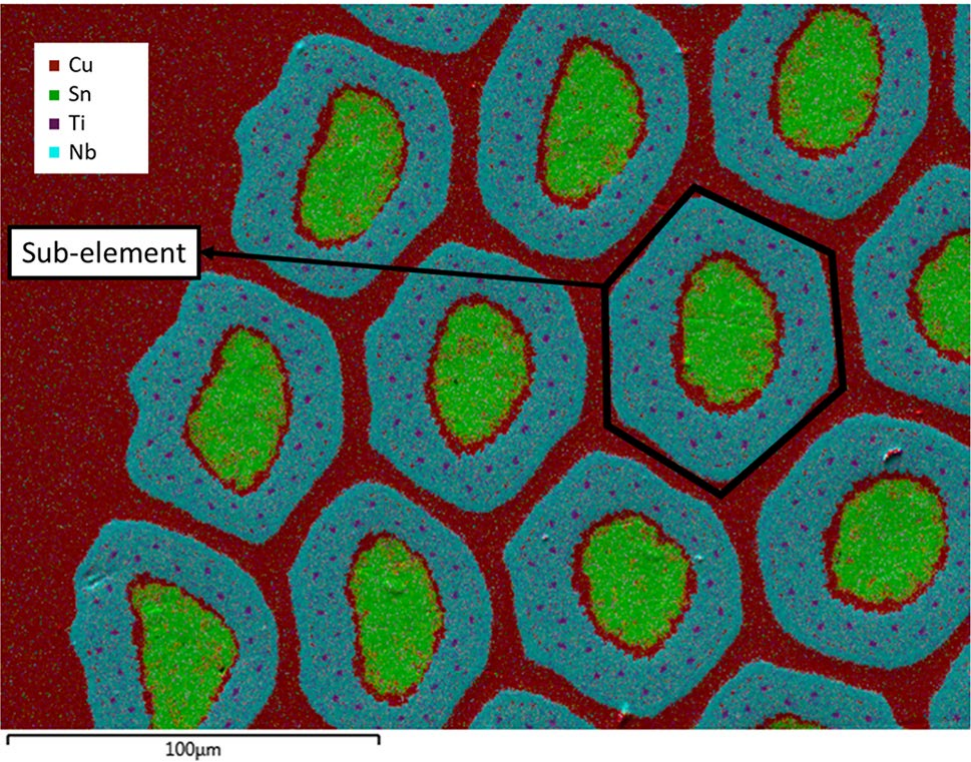
Cross section of S21 Nb_3_Sn RRP wire before heat treatment. The picture is a combination of a Scanning Electric Microscope (SEM) image and an Energy-dispersive X-ray spectroscopy (EDX) analysis of the surface. The sub-elements are surrounded by high purity Cu matrix. Each sub-element is an assembly of Nb and Nb-Ti filaments in a Cu matrix built around a Sn rod. The Nb barrier, around the sub-element, prevents Sn diffusion in the Cu matrix during the heat treatment.

RRPs are a type of Internal-Tin Nb_3_Sn wires, whose repetitive unit, called sub-element, is made of Nb filaments in a Cu matrix around a Sn core, the whole assembly being surrounded by a Nb barrier and then an outer layer of pure Cu. The RRP wire is then produced stacking and cold drawing several sub-elements in high purity Cu tube (sometimes with additional pure Cu hexagonal rods in the center) down to the wire final size, see Fig. 10. The Nb barrier is intended to prevent the Sn diffusion from the sub-element into the high purity Cu matrix, which is the reason why RRP are also called distributed-barrier wires^60^. The superconducting compound is formed during a tailored heat treatment which activates the Nb-Sn reaction. The Nb-Sn compound is superconducting between 18at% Sn and 25at% Sn, reaching the highest *T*_*c*_ and *B*_*c2*_ when the compound is about 24–24.4%, i.e. Nb_3_Sn. Nevertheless, stoichiometric ratio is generally avoided in the wires, as could result in a complete reaction of the barrier, with consequent Sn leakage. Typically a Nb:Sn molar ratio from 3.1 to 3.6 is used to guarantee a supply of Sn that is sufficient to fully react all the Nb filaments, but without an excess that would take valuable “real estate” in the wire and would lead to a too fast growth of Nb_3_Sn from the diffusion barrier Nb and thus beat the purpose of the barrier itself^61^. It has been shown in^42^ that a Nb:Sn molar ratio of 3.6 greatly enhances the Residual-Resistivity-Ratio (RRR) of the wire compared to lower Nb:Sn values. The RRR is used as indication of the purity of the Cu matrix. It is defined as the ratio of the Cu matrix resistivity at room temperature to its resistivity above the superconducting transition. In case of superconducting wires for high field magnets, Cu with high RRR is necessary for electro-thermal stability^62^ and a low RRR can indicate damages of the Nb barrier and Sn leakage into the Cu matrix.

In order to achieve high *J*_*c*_, RRP production process includes a very low Cu content inside the sub-elements causing proximity between the Nb filaments^63^. In the heat treatment, the volume expansion during the Nb_3_Sn formation causes the coalescence of the filaments and the formation of a monolithic Nb_3_Sn ring, corresponding to filament effective diameter (*d*_*eff*_) of 30–70 µm. Large *d*_*eff*_ can generate issues in application that requires low hysteresis loss and high magnetic field quality as in accelerator magnets^64^.

The design of RRP wires is defined by the number of restacked sub-elements. Sub-elements are deformed to hexagonal shape and they are stacked using a centered hexagonal grid. As said, high purity Cu is necessary for electro-thermal stability of the wire. The Cu matrix has double role: it acts as low resistance current shunt in case of transition of the superconductor to normal state and it homogenizes the superconductor temperature because its thermal conductivity is orders of magnitude higher than that of Nb_3_Sn. The amount of Cu present in a wire is defined using the ratio between Cu and non-Cu materials, so called Cu:Non-Cu ratio. This ratio is tailored depending on the wire application. In RRP wires, the Cu included in the sub-elements dissolves in the Sn core so it cannot be considered as assisting the stability, therefore to reach the desired Cu:non-Cu ratio, usually between 1 and 1.5^62^, some of the wire sub-elements must be substituted by high purity Cu hexagons. In this way, an additional number is given for the wire definition, which is the number of the total superconductive sub-elements. For example, a 132/169 wire consists of 169 restacked sub-elements of which 132 are Nb_3_Sn.

### RRR measurements

The RRR sample holder is designed to test eight straight samples per measurement. The samples are mounted in series and they are soldered to the current leads. The voltage-tap pairs are soldered on each sample separated by about 20 mm. The resistance (R) at room temperature is measured injecting in the samples a current of 1 A and measuring the voltage drops trough the taps. For the resistance at cryogenic temperature, the sample holder is mounted on a probe, which is inserted in a cold cryostat and gradually lowered to cool the samples up to liquid helium temperature. A thermometer is placed on the sample holder to monitor the temperature. A current of 10 A was injected through the samples and the voltage across each wire was measured to determine the resistance. The cryostat is then slowly heat up, increasing steadily the temperature. The resistance is measured as function of the temperature from 4.2 K up to 25 K, which is after the Nb_3_Sn transition. RRR is defined as the ratio between R at room temperature and at 18 K, which is just after the transition to normal state.

### Electron microscopy description

Electron microscopy is performed using a JEOL JSM-7600F field emission scanning electron microscope (FESEM). Energy-dispersive X-ray spectroscopy (EDS) is performed in the FESEM using an Oxford Instrument X-Max system, which utilizes a large area analytical Silicon Drift EDS Detector (SDD) with PentaFET Precision, at an acceleration voltage of 16 kV.
